# Major predictors and management of small-bowel angioectasia

**DOI:** 10.1186/s12876-015-0337-8

**Published:** 2015-08-25

**Authors:** Atsushi Igawa, Shiro Oka, Shinji Tanaka, Sayoko Kunihara, Makoto Nakano, Taiki Aoyama, Kazuaki Chayama

**Affiliations:** 1 Department of Gastroenterology and Metabolism, Graduate School of Biomedical Sciences, Hiroshima University, Hiroshima, Japan; 2 Department of Endoscopy, Hiroshima University, 1-2-3 Kasumi, Minami-ku, Hiroshima 734-8551 Japan

## Abstract

**Background:**

Small-bowel angioectasias are frequently diagnosed with capsule endoscopy (CE) or balloon endoscopy however, major predictors have not been defined and the indications for endoscopic treatment have not been standardized. The aim of this study was to evaluate the predictors and management of small-bowel angioectasia.

**Methods:**

Among patients with obscure gastrointestinal bleeding (OGIB) who underwent both CE and double-balloon endoscopy at our institution, we enrolled 64 patients with small-bowel angioectasia (angioectasia group) and 97 patients without small-bowel angioectasia (non-angioectasia group). The angioectasia group was subdivided into patients with type 1a angioectasia (35 cases) and type 1b angioectasia (29 cases) according to the Yano-Yamamoto classification. Patient characteristics, treatment, and outcomes were evaluated.

**Results:**

Age (*P* = 0.001), cardiovascular disease (*P* = 0.002), and liver cirrhosis (*P* = 0.003) were identified as significant predictors of small-bowel angioectasia. Multivariate logistic regression analysis identified cardiovascular disease (odds ratio 2.86; 95 % confidence interval, 1.35–6.18) and liver cirrhosis (odds ratio 4.81; 95 % confidence interval, 1.79–14.5) as independent predictors of small-bowel angioectasia. Eleven type 1a cases without oozing were treated conservatively, and 24 type 1a cases with oozing were treated with polidocanol injection (PDI). Re-bleeding occurred in two type 1a cases (6 %). Seventeen type 1b cases were treated with PDI and 12 type 1b cases were treated with PDI combined with argon plasma coagulation (APC) or clipping. Re-bleeding occurred in five type 1b cases (17 %) that resolved after additional endoscopic hemostasis in all cases. There was one adverse event from endoscopic treatment (1.6 %).

**Conclusions:**

Cardiovascular disease and liver cirrhosis were significant independent major predictors of small-bowel angioectasia. Type 1a angioectasias with oozing are indicated for PDI and type 1b angioectasias are indicated for PDI with APC or clipping.

## Background

Small-bowel vascular lesions, especially small-bowel angioectasias, account for a large number of cases of obscure gastrointestinal bleeding (OGIB) [1]. Small-bowel bleeding accounts for 5 % of all gastrointestinal bleeding cases [2], and vascular lesions account for 23–52 % of cases of small-bowel bleeding [3–5]. Small-bowel angioectasias comprise the majority of small-bowel vascular lesions and are found in 30–40 % of OGIB cases [6]. Despite these facts, the major predictors for small-bowel angioectasia have not been determined.

**Fig. 1:**
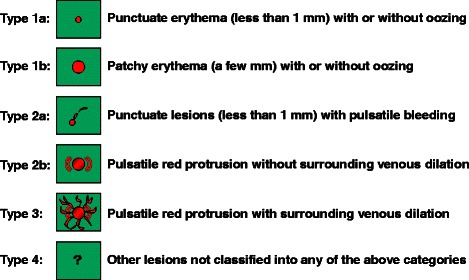
Endoscopic classification of small-bowel vascular lesions (Yano-Yamamoto classification)

Angioectasia is a collection of abnormal blood vessels composed of thin tortuous capillaries without an internal elastic membrane. Yano-Yamamoto’s [7] accepted endoscopic classification of small-bowel vascular lesions classifies small-bowel angioectasia as a type 1 lesion (Fig. 1). These lesions are further subclassified, as follows: type 1a lesions are characterized by punctate erythema (<1 mm) with or without oozing, and type 1b lesions are characterized by patchy erythema (2–3 mm) with or without oozing. Fan et al. reported the use of argon plasma coagulation (APC) for the endoscopic treatment of small-bowel angioectasia [8]. However, there is no consensus on the optimal endoscopic treatment of small-bowel angioectasia.

We analyzed patient characteristics and endoscopic treatments for small-bowel angioectasia cases diagnosed using both capsule endoscopy (CE) and double-balloon endoscopy (DBE) at our institution and followed for at least 1 year. The aim of this study was to determine the major predictors and optimal endoscopic treatment of small-bowel angioectasia.

## Methods

### Patients

**Fig. 2:**
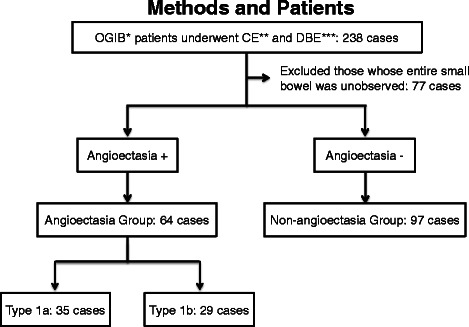
Flow chart of study patients. * Obscure gastrointestinal bleeding ** Capsule endoscopy *** Double-balloon endoscopy

A total of 800 patients with OGIB presented to Hiroshima University Hospital between April 2004 and December 2013. Two hundred thirty-eight of those patients underwent both CE and DBE. Sixty-four patients diagnosed with small-bowel angioectasia were assigned to the angioectasia group. Ninety-seven patients without small-bowel angioectasia were assigned to the non-angioectasia group. We excluded 77 patients in whom the entire small bowel had not been visualized. The angioectasia group was subclassified into patients with type 1a (35 cases) and type 1b (29 cases) angioectasias according to the Yano-Yamamoto classification (Fig. 2). All patients underwent upper and lower gastrointestinal endoscopies prior to CE and DBE.

The study was conducted in accordance with the Declaration of Helsinki and was approved by the Institutional Review Board of Hiroshima University Hospital. Written informed consent was obtained from all of the patients who participated in the study.

### CE procedure

CE was performed using PillCam SB1 or PillCam SB2 capsules (Given Imaging Ltd, Yokneam, Israel). The capsule was swallowed with a dimethicone solution after an overnight fast, without any other preparation. Patients were allowed to drink clear liquids and eat a light meal 2 h and 4 h after swallowing the capsule, respectively. The sensor array and recording device were removed 8 h after swallowing the capsule. Images were analyzed with Rapid Reader 6 software on a RAPID 5 or 6.5work station (software and workstation from Given Imaging Ltd).

### DBE procedure

All DBE procedures were performed using the DBE system (FUJIFILM, Saitama, Japan) employing a FUJIFILM EN-450 P5 or EN-450 T5-type endoscope. Per oral insertion of the endoscope required that patients fast for 8–12 h, similar to the preparation for upper gastrointestinal endoscopy. All rectal insertions required a preparation similar to that used for colonoscopy. Patients were lightly sedated with pentazocine (15 mg) and midazolam (0.05 mg/kg). We selected the oral or rectal route based on the results of CE. If a source of bleeding was not identified during the examination, we marked the small-bowel mucosa with pure carbon at the most distal point.

### Strategy of endoscopic treatment for small-bowel angioectasia

Type 1a lesions without oozing were treated conservatively without any endoscopic intervention. Type 1a lesions with oozing were treated with 1 % polidocanol injection (PDI) in all cases. Type 1b lesions were treated with PDI or PDI combined with APC or clipping, according to the condition of the lesions or the endoscope used for treatment. We analyzed the re-bleeding rate and endoscopic treatment related adverse events. Re-bleeding was defined as evidence of recurrent visible gastrointestinal bleeding (melena or hematochezia) with recent negative upper and lower gastrointestinal endoscopies and/or a subsequent decrease in the hemoglobin level by >2 g/dL from baseline. The definition of re-bleeding also included small-bowel bleeding from lesions other than those previously treated by endoscopic hemostasis. In addition, follow-up CE or DBE was performed 3–6 months after endoscopic hemostasis for all patients.

### Evaluation

Patient characteristics, including age, sex, bleeding type (overt or occult), drug use (antithrombotic and nonsteroidal anti-inflammatory drugs), and underlying diseases (cerebrovascular disease, cardiovascular disease, chronic renal failure, hypertension, dyslipidemia, diabetes, and liver cirrhosis) were recorded for the two groups. Lesion characteristics for the type 1a and type 1b groups, including number of lesions, location in the small-bowel, outcomes after endoscopic treatment, and re-bleeding rate were evaluated. In addition, we divided the type 1b group into two groups according to the treatment methods used (treated with PDI or treated with PDI combined with APC or clipping). The rate of re-bleeding-free survival was calculated and compared between the two groups. The entire small bowel was divided into three parts (upper, middle, and lower) based on CE transit times in accordance with a previous report by Li et al [9]. The first two parts of the small bowel were considered the jejunum, and the last part was considered the ileum.

### Statistical analysis

Comparisons were performed using the unpaired *t*-test for quantitative data and the chi-squared test for categorical data. A Yates correction or Fisher’s exact test was used when required. All tests were two-sided, and a *P*-value < 0.05 was considered statistically significant. Odds ratios (ORs) and 95 % confidence intervals (95 % CIs) were used in the multivariate logistic regression analyses. The re-bleeding-free interval was estimated using the Kaplan-Meier method. The JMP10 statistical software package (SAS Institute Inc., Cary, NC, USA) was used for all analyses.

## Results

Patient age, cardiovascular disease, and liver cirrhosis were significantly associated with small-bowel angioectasia (*P* = 0.001, *P* = 0.002, and *P* = 0.003, respectively) (Table 1). On multivariate logistic regression analysis, cardiovascular disease (OR, 2.86; 95 % CI, 1.35–6.18) and liver cirrhosis (OR, 4.81; 95 % CI, 1.79–14.5) were significant predictors of small-bowel angioectasia (Table 2). In addition, a separate analysis of the type 1a and type 1b groups revealed no differences between the two groups (Table 3).

**Table 1:** Characteristics of patients in the angioectasia and non-angioectasia groups

Patient characteristics	Angioectasia group	Non-angioectasia group	*P*-value
	(*n* = 64)	(*n* = 97)	
Sex			
Male	39	57	n.s
Female	25	40	
Age (years)			
Mean ± SD	71.2 ± 13.9	63.4 ± 17.3	<0.01
Median (range)	75.5 (27–87)	66 (17–85)	
< 65	18	43	<0.05
≧65	46	54	
OGIB type			
Occult	15	20	n.s
Overt	49	77	
Drug use			
Antithrombotic			
Yes	20	18	n.s
No	44	79	
NSAIDS			
Yes	6	20	n.s
No	58	77	
Underlying disease			
Cerebrovascular disease			
Yes	6	8	n.s
No	58	89	
Cardiovascular disease			
Yes	27	19	<0.01
No	37	78	
Chronic renal failure			
Yes	6	7	n.s
No	58	90	
Hypertension			
Yes	38	43	n.s
No	26	54	
Dyslipidemia			
Yes	9	10	n.s
No	55	87	
Diabetes			
Yes	15	16	n.s
No	49	81	
Liver cirrhosis			
Yes	15	6	<0.01
No	49	91	

*Abbreviations: OGIB* obscure gastrointestinal bleeding; *NSAIDS* nonsteroidal anti-inflammatory drugs

**Table 2:** Multivariate logistic regression analyses of predictors of small-bowel angioectasia

Clinical factors	Odds ratio	95 % confidence interval	*P*-value
Liver cirrhosis	4.81	1.79–14.5	0.0028
Cardiovascular disease	2.86	1.35–6.18	0.0066
Age (≧65)	1.25	0.60–2.60	0.550

**Table 3:** Comparison of patient’s characteristics between type 1a and 1b angiectasias

Patient characteristics	Angioectasia		*P*-value
	Type 1a (*n* = 35)	Type 1b (*n* = 29)	
Sex			
Male	22	17	n.s
Female	13	12	
Age (years)			
Mean ± SD	69.3 ± 16.4	73.5 ± 10.8	n.s
Median (range)	75 (27–87)	78 (42–86)	
< 65	12	6	n.s
≧65	23	23	
OGIB type			
Occult	9	6	n.s
Overt	26	23	
Drug use			
Antithrombotic			
Yes	10	10	n.s
No	25	19	
NSAIDS			
Yes	3	3	n.s
No	32	26	
Underlying disease			
Cerebrovascular disease			
Yes	3	3	n.s
No	32	26	
Cardiovascular disease			
Yes	14	13	n.s
No	21	16	
Chronic renal failure			
Yes	3	3	n.s
No	32	26	
Hypertension			
Yes	18	19	n.s
No	17	10	
Dyslipidemia			
Yes	4	5	n.s
No	31	24	
Diabetes			
Yes	7	7	n.s
No	28	22	
Liver cirrhosis			
Yes	8	7	n.s
No	27	22	

*Abbreviations: OGIB* obscure gastrointestinal bleeding; *NSAIDS* nonsteroidal anti-inflammatory drugs

**Fig. 3:**
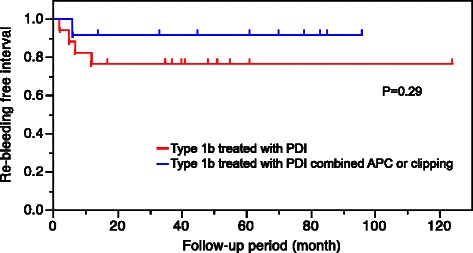
Kaplan-Meier curve for post treatment re-bleeding according to treatment for patients with type 1b angioectasia. PDI, polidocanol injection; APC, argon plasma coagulation

Eleven type 1a cases without oozing were treated conservatively without endoscopic intervention, and 24 type 1a cases with oozing were treated endoscopically with PDI. Re-bleeding occurred in two cases (6 %), one was treated conservatively and the other was treated with PDI. The mean time to re-bleeding was 23 days, and in both cases, bleeding occurred from lesions other than those previously treated. One patient had bleeding from a small-bowel arteriovenous malformation and the other had a small-bowel Dieulafoy’s lesion. Of the type 1b cases, 17 were treated with PDI and 12 were treated with PDI combined with APC or clipping. Re-bleeding occurred in five cases (17 %), four who had been initially treated with PDI, and one who had been initially treated with PDI combined with APC (Table 4). The mean time to re-bleeding was 123 days. Four re-bleeding cases involved previously treated lesions, and one involved a previously untreated lesion. Re-bleeding was controlled conservatively with additional endoscopic hemostasis in all cases. The Kaplan-Meier curve for post-treatment re-bleeding in type 1b angioectasias showed no statistically significant difference between those treated with PDI alone and those treated with PDI combined with APC or clipping (*P* = 0.29) (Fig. 3). One adverse event (1.6 %) occurred as a result of endoscopic treatment in a patient who developed an ulcer after PDI. This patient recovered with conservative treatment.

**Table 4:** Comparison of lesion characteristics between type 1a and 1b angioectasias

Lesion characteristics	Angioectasia		*P*-value
	Type 1a (*n* = 35)	Type 1b (*n* = 29)	
Number			
Single	20	18	n.s
Multiple	15	11	
Localization of the small-bowel			
Upper	12	13	n.s
Middle	12	10	
Lower	11	6	
First treatment			
Follow up	11	0	
PDI	24	17	
PDI + APC or clipping	0	12	
Re-bleeding after treatment, n (%)	2 (6 %)	5 (17 %)	n.s
Present lesion	0	4	
New other lesion	2	1	
Days to re-bleeding	23	123	
Follow-up period, mean ± SD [months]	54 ± 30	53 ± 25	n.s

Abbreviations: *PDI* polidocanol injection, *APC* argon plasma coagulation

## Discussion

The etiology of gastrointestinal angioectasia remains unclear. Regula et al. suggested that a neurohormonal abnormality, in which sympathetic nerve stimulation occurs in response to chronic hypoperfusion, leads to relaxation of intestinal vascular smooth muscle, causing local vascular overload, dilation, and eventually, permanent angioectasia [10]. Junquera et al. reported that the overexpression of angiogenic factors, such as basic fibroblast growth factor and vascular endothelial growth factor, seems to play a pathogenic role in the development of angioectasia [11, 12].

In this study, cardiovascular disease and liver cirrhosis were independent predictors of small-bowel angioectasia. Aortic stenosis [13], chronic renal failure [14], and von Willebrand disease [15] have all been associated with gastrointestinal angioectasia. Hemorrhagic gastrointestinal angioectasia associated with aortic stenosis has been reported as Heyde’s syndrome [13]. In the present study, there were four patients in the angioectasia group (6 %) who had aortic stenosis. Additionally, portal hypertensive enteropathy (PHE) associated with portal hypertension in patients with liver cirrhosis has been reported to be a major cause of gastrointestinal bleeding [16]. The endoscopic findings for PHE have been described as red spots, erosions, angioectasia, villous edema, and varices. The presumed cause is blood-flow stasis associated with portal hypertension however, the pathogenesis remains unknown. De Palma et al. conducted a CE-based study and found PHE in 67 % of patients with liver cirrhosis [17]. We previously reported the use of CE to diagnose PHE, especially in patients with Child-Pugh class B or C, ascites, and portal hypertensive gastropathy in patients with compensated liver cirrhosis and portal hypertension [18]. In addition, previously reported major predictors of PHE were the presence of a left gastric vein and splenorenal shunts [19]. Therefore, we believe that patients with OGIB with cardiovascular disease and/or liver cirrhosis should undergo CE and/or DBE to evaluate possible bleeding from small-bowel angioectasia.

The clinical usefulness of CE for the evaluation of OGIB has been previously reported [20, 21], and CE has become the initial diagnostic choice for the evaluation of patients with OGIB who do not require emergency hemostatic intervention. Small-bowel angioectasias may be easily missed by CE because of their small size; however, we have reported increased detection rates with the use of flexible spectral imaging color enhancement (FICE) [22, 23]. Maeda et al. also reported that FICE 3 was useful for the differential diagnosis of red spots and angioectasia in the small-bowel [24]. In addition, we compared the diagnostic capability of CE and DBE to detect small-bowel lesions in patients with OGIB who had their entire small bowel visualized. The diagnostic concordance rates for CE and DBE were high for small-bowel lesions, and when results varied, it was due to false-negative CE results [25]. Postga et al. reported that false- negative cases with CE were more typically seen with upper small-bowel lesions [26]. Detailed observation is necessary, and repeat CE or DBE should be performed when bleeding persists despite a previously negative CE results in patients with OGIB.

Endoscopic injection sclerotherapy with polidocanol has been used for endoscopic hemostasis in patients with esophageal varices [27] and gastric ulcers with excellent results [28]. Okano et al. reported the early and late hemostatic properties of polidocanol from research conducted in dogs. The early hemostatic effects were the result of pressure on the blood vessels with associated interstitial edema and thrombosis formation in small blood vessels, and the late hemostatic effects were due to thrombus formation from vascular inflammation [29]. Takeuchi et al. reported that the effects of PDI were limited to the submucosal layer and that deeper penetration and perforation after PDI did not occur [30].

Re-bleeding in patients with type 1a angioectasia was secondary to other vascular lesions that had not been previously treated. As there is a possibility of missing small lesions, such as Dieulafoy’s lesions, in the small bowel, we performed a detailed follow-up observation because of the possibility of heterochronic multiple small-bowel vascular lesions. In contrast, re-bleeding occurred in five patients with type 1b (17 %), and, in four of these cases, bleeding occurred in previously treated lesions. Fan et al. reported that the rate of re-bleeding from previously treated small-bowel angioectasias was about 25 % and that advanced age was a risk factor for re-bleeding [8]. Rahmi et al. also reported in a long-term follow-up study that cardiovascular disease was an independent risk factor for re-bleeding in patients with OGIB with small-bowel angioectasia treated by DBE [31]. In the present study, endoscopic treatments successfully treated type 1a angioectasias. Type 1b angioectasias treated with PDI were more likely to have re-bleeding compared to type 1b angioectasias treated with PDI combined with APC or clipping, but the difference was not statistically significant, likely due to the small number of cases. PDI combined with APC or clipping should be considered in type 1b angioectasia because PDI alone may not be efficacious. Therefore, it appears that the appropriate endoscopic treatment method is determined according to the type of small-bowel angioectasia. In addition, since all re-bleeding cases occurred within 1 year, we recommend strict observation for 1 year after endoscopic treatment.

Our study had some limitations. First, it was a retrospective study, and therefore, a selection bias was inevitable. Second, the study included patients from a single center only. Therefore, a large-scale, prospective study is needed to address these limitations.

## Conclusions

Cardiovascular disease and liver cirrhosis were significant independent major predictors of small-bowel angioectasia. In the management of small-bowel angioectasia, type 1a lesions without oozing do not require endoscopic treatment. However type 1a angioectasias with oozing are indicated for PDI, and type 1b angioectasias are indicated for PDI combined with APC or clipping.

## Abbreviations

APC: Argon plasma coagulation
CE: Capsule endoscopy
CI: Confidence interval
DBE: Double-balloon endoscopy
FICE: Flexible spectral imaging color enhancement
OGIB: Obscure gastrointestinal bleeding
OR: Odds ratio
PDI: Polidocanol injection
PHE: Portal hypertensive enteropathy
